# Respirators in Healthcare: Material, Design, Regulatory, Environmental, and Economic Considerations for Clinical Efficacy

**DOI:** 10.1002/gch2.202200001

**Published:** 2022-04-12

**Authors:** Cameron C. Young, James D. Byrne, Adam J. Wentworth, Joy E. Collins, Jacqueline N. Chu, Giovanni Traverso

**Affiliations:** ^1^ Division of Gastroenterology Brigham and Women's Hospital Harvard Medical School 75 Francis St Boston MA 02115 USA; ^2^ Departments of Chemical Engineering and Biochemistry Northeastern University 300 Huntington Ave Boston MA 02115 USA; ^3^ Harvard Radiation Oncology Program 55 Fruit St Boston MA 02114 USA; ^4^ David H. Koch Institute for Integrative Cancer Research Massachusetts Institute of Technology 500 Main St. Building 76 Cambridge MA 02142 USA; ^5^ Department of Mechanical Engineering Massachusetts Institute of Technology 77 Massachusetts Ave Cambridge MA 02139 USA; ^6^ Department of Radiation Oncology Dana‐Farber Cancer Institute/Brigham and Women's Hospital 44 Binney St Boston MA 02115 USA; ^7^ Division of Gastroenterology Massachusetts General Hospital 55 Fruit St Boston MA 02114 USA

**Keywords:** airborne hazards, N95 masks, occupational health, respirators, respiratory protection

## Abstract

Maintaining an ample supply of personal protective equipment continues to be a challenge for the healthcare industry, especially during emergency situations and times of strain on the supply chain. Most critically, healthcare workers exposed to potential airborne hazards require sufficient respiratory protection. Respirators are the only type of personal protective equipment able to provide adequate respiratory protection. However, their ability to shield hazards depends on design, material, proper fit, and environmental conditions. As a result, not all respirators may be adequate for all scenarios. Additionally, factors including user comfort, ease of use, and cost contribute to respirator effectiveness. Therefore, a careful consideration of these parameters is essential for ensuring respiratory protection for those working in the healthcare industry. Here respirator design and material characteristics are reviewed, as well as properties of airborne hazards and potential filtration mechanisms, regulatory standards of governmental agencies, respirator efficacy in the clinical setting, attitude of healthcare personnel toward respiratory protection, and environmental and economic considerations of respirator manufacturing and distribution.

## Graphical abstract

Healthcare workers exposed to potential airborne hazards require sufficient respiratory protection. The ability of respirators to shield hazards depends on design, material, proper fit, and environmental conditions. Here respirator design and material characteristics are reviewed, as well as properties of airborne hazards and filtration mechanisms, regulatory standards, respirator efficacy in clinical settings, attitudes toward respiratory protection, and environmental and economic considerations.

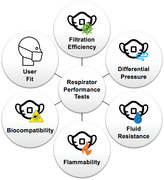

## 1 Introduction

Effective respiratory protection is essential for healthcare workers, as it prevents exposure to infectious diseases and other occupational hazards. Personal protective equipment (PPE) remains the most effective way to limit exposure when engineering and administrative controls fail. The clinical environment contains a disproportionately large number of airborne hazards; therefore, institutions must maintain adequate supplies of respiratory protection and devise strategies to ensure worker safety. Widespread shortages of respirators and other PPE during the recent coronavirus disease 2019 (COVID‐19) pandemic has underscored this need.^[^ ^1^ ^]^ Throughout history, humans have identified the need for respiratory protection and, over time, have improved respirator technology and increased its efficacy. Research has shown that respirator effectiveness is highly dependent on a number of factors including filtration efficiency of the filter media, adequacy of fit, and user compliance.^[^ ^2^, ^3^, ^4^ ^]^ Therefore, modern respirator design requires a careful understanding and consideration of these characteristics to ensure efficacy.

Respirators come in a wide range of designs and shapes, with disposable and non‐disposable varieties, and have various coverage options: some cover just the nose and mouth, some cover the entire face, and some can form an isolated environment with supplied air. Furthermore, non‐woven, layered filter media is employed in many disposable respirators, while reusable respirators frequently feature an elastomeric design with replaceable filters or cartridges. Filter media and respirator cartridges are manufactured to capture airborne particles through a number of filtration strategies that exploit the natural behavior of particulate matter. Although a range of materials and respirator designs may be effective, governmental regulatory agencies like the National Institute for Occupational Safety and Health (NIOSH), the Occupational Health and Safety Administration (OSHA), and the Food and Drug Administration (FDA) set minimum standards for respirator performance to ensure worker safety. These agencies evaluate respirators for several parameters including filtration efficiency, differential pressure drop, fluid resistance, flammability, biocompatibility, and user fit. Successful performance in these evaluations is required to receive approval and certification. Additionally, in the healthcare industry, employee attitudes towards respiratory protection, education, user comfort, and ease of use play a role in the effectiveness of respirators. Institutions must also consider the environmental, economic, and logistical implications of supplying respiratory protection to their employees. In this review, we evaluate the design and material characteristics of respirators, regulatory standards of governmental agencies, efficacy of respirators in the clinical setting, and other environmental, economic, and logistical considerations of respiratory protection.

## 2 History

The dangers of inhaled toxins have long been known and various protection strategies have been developed to mitigate their harm. The Romans utilized a rudimentary mask made of animal bladder to protect themselves from airborne hazards during mining; this eventually evolved into the use of sackcloth filters.^[^ ^5^ ^]^ More recently, the expanded mining practices in the United States during the 19th century resulted in numerous occupational deaths, not only from mining collapses and disasters, but also from coal workers’ pneumoconiosis or “black lung disease,” a respiratory illness characterized by pulmonary inflammation and fibrosis caused by the inhalation of coal dust.^[^ ^6^ ^]^ Similarly, firefighters during this time were frequently exposed to smoke inhalation and experienced a critical need for respiratory protection.^[^ ^7^ ^]^ In an effort to mitigate mining‐related fatalities and expand protections for workers, the United States Department of the Interior established the United States Bureau of Mines (USBM) to regulate mining practices and improve worker safety. This organization spearheaded modern respirator research and design which led to the development of self‐contained breathing apparatuses, half‐ and full‐face masks, and respirators with removable filter cartridges. According to initial USBM guidelines, respiratory equipment, were required to meet the following criteria: provide adequate protection, be reasonably comfortable and physically convenient, protect the user for an acceptable amount of time, and be constructed of durable material. The first self‐contained breathing apparatus for mine rescue was approved by the USBM in 1919.^[^ ^8^ ^]^ USBM regulations gradually evolved over time to include guidance for the approval of gas masks, particulate filtering respirators, and chemical cartridge respirators; this organization eventually branched into NIOSH in 1970 which took over responsibility for occupational respirator evaluation and approval.^[^ ^8^ ^]^

The use of respiratory protection in healthcare dates back to the late 19th century when German scientist Carl Flügge developed the droplet theory of disease transmission in 1899.^[^ ^9^ ^]^ During the early 20th century, several scientists and clinicians began to recognize the dangers of respiratory disease transmission and suggested the use of masks by healthcare workers. Early surgical masks consisted simply of roller gauze placed over the nose and mouth. Widespread mask use in the healthcare industry began following World War I, where the transmission of respiratory illnesses among soldiers and caretakers was common. One report during this era showed that consistent mask use by clinicians could decrease infection rates by as much as 95%, prompting wide adoption of mask‐wearing across the industry after the war.^[^ ^10^ ^]^ Several peacetime studies continued to show that the use of respiratory protection could prevent infections in clinicians and patients, which prompted the development of more convenient and durable mask designs, with paper masks being introduced in the 1930s and masks made of synthetic materials reaching the market in the 1960s.^[^ ^11^ ^]^ The N95 respirator was initially designed for industrial use and was approved by NIOSH in 1972. These respirators entered the healthcare industry in the 1980s due to the increased prevalence of *Mycobacterium tuberculosis* infections in the United States.^[^ ^12^ ^]^ More recently, electrostatic virus‐blocking technology was developed in 1995, which eventually evolved into the modern N95 respirator.^[^ ^13^ ^]^ The COVID‐19 pandemic has presented unprecedented challenges for the healthcare industry and has strained the respirator supply chain; therefore, new research and advancements are underway to continue to improve respirator design for healthcare use.

## 3 Overview of Respirator Types

**Figure 1:**
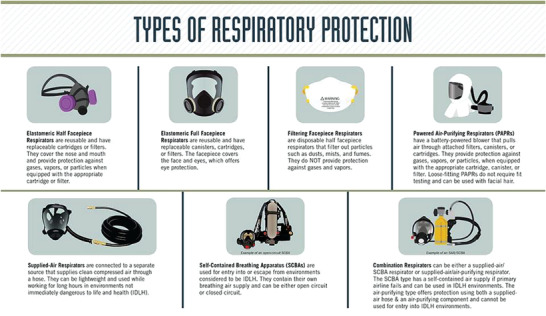
Types of respiratory protection. Reproduced with permission.^[^ ^15^ ^]^ Copyright 2019, Centers for Disease Control and Prevention, National Institute for Occupational Health and Safety.

Respirators are broadly classified into two categories: air‐purifying respirators (APRs), which purify ambient air before supplying it to the user, and atmosphere‐supplying respirators, which directly deliver a clean air supply isolated from the surrounding environment (**Figure** **1**).^[^ ^14^, ^15^ ^]^ Atmosphere‐supplying respirators are required for use in environments considered oxygen‐deficient^[^ ^16^ ^]^ or immediately dangerous to life or health,^[^ ^17^ ^]^ and are therefore uncommonly encountered in the healthcare industry. APRs, however, are vital pieces of PPE found throughout hospitals and other clinical settings. APRs can be further divided into three classifications: filtering facepiece respirators (FFRs), elastomeric half facepiece respirators (EHFRs), and powered APRs (PAPRs).^[^ ^18^ ^]^

FFRs function by filtering aerosols and other particles directly through filter media, which forms a barrier between the user's respiratory system and the environment during the active breathing process. These respirators are fitted tightly around the wearer's nose and mouth, forming a seal that forces inhaled air through the filter media. FFRs are certified and rated by NIOSH for filtration capabilities and oil resistance. The nine classes of respirators defined by NIOSH include N (not resistant to oil), R (resistant to oil), and P (oil‐proof), at filtration efficiencies of 95, 99, and 100, corresponding to particle filtration efficiencies (PFEs) of at least 95%, 99%, and 99.97%, respectively.^[^ ^19^ ^]^ The N95 certification is the least stringent as well as the most commonly used respirator for commercial, industrial, and healthcare use.^[^ ^20^ ^]^ As proper fit is essential to the functioning of FFRs, OSHA requires all individuals that wear these respirators in an occupational setting to undergo annual fit testing.^[^ ^16^, ^21^ ^]^ Proper use of FFRs is able to reduce workplace exposures of airborne particles to 1/10th of the concentration in the air, corresponding to an OSHA‐assigned protection factor (APF) of 10.^[^ ^22^ ^]^ Current recommendations advise that N95 respirators be used only once prior to replacement in most non‐emergency situations.^[^ ^23^ ^]^ However, in times of widespread PPE shortage, it may be necessary to use N95 respirators for an extended period of time or reuse them for a limited number of times.^[^ ^24^ ^]^ Novel strategies to perform large‐scale respirator decontamination^[^ ^25^ ^]^ and develop reusable N95 respirators are currently under investigation.^[^ ^26^, ^27^ ^]^

EHFRs differ from FFRs in a number of ways; most notably, they are reusable. An EHFR is comprised of two components: a facepiece made of silicon or rubber, and exchangeable cartridges that filter and purify air. The elastomeric facepiece forms a tight seal around the user's nose and mouth, forcing all inhaled air through the cartridges during the active breathing process. These replaceable cartridges contain a combination of filters, sorbents, and other materials that can purify ambient air from aerosols, particles, vapors, gases, and other contaminants. Specific cartridges are effective against specific agents; therefore, these can be selected and changed to combat unique environmental hazards. EHFRs should be sanitized after each use and the cartridges should be replaced frequently to ensure proper functioning, according to OSHA regulations and the manufacturer's guidance.^[^ ^16^ ^]^ EHFRs contain two filtration cartridges, while elastomeric quarter facepiece respirators contain only one filtration cartridge while still covering the nose and mouth. Both of these elastomeric respirators are able to achieve an OSHA APF of 10.^[^ ^22^ ^]^ Elastomeric full facepiece respirators feature two exchangeable filtration cartridges, but offer complete coverage of the entire face, including the eyes, nose, and mouth, with an OSHA APF of up to 50 when worn properly.^[^ ^22^ ^]^ Each of these elastomeric respirators, like FFRs, requires annual fit testing for proper use.^[^ ^21^ ^]^

PAPRs are a unique class of respirators that feature an active, battery‐powered system that forces ambient air through a filtration system before delivering it to the user. PAPR filters contain high‐efficiency cartridges similar to EHFRs that are effective in removing aerosols, particles, vapors, gases, and other contaminants. These respirators can be either the full‐ or half‐facemask varieties and are frequently accompanied by a body substance isolation suit. As such, PAPRs can deliver purified air directly to the wearer's nose and mouth, or supply it to the isolated environment, reducing breathing resistance. Positive pressure provided by these powered systems increases protection by reducing leakage of ambient air into the user's breathing environment. As the most comprehensive and isolated respirator systems, PAPRs have the highest OSHA APF, with a value of 50 for half facepiece PAPRs and up to 1000 for full facepiece PAPRs.^[^ ^22^ ^]^

## 4 Evaluation of Respiratory Protection in the Healthcare Industry

FFRs play a critical role in the healthcare industry, where the demand, for number of respirators consumed annually, is five times greater than any other industry.^[^ ^28^ ^]^ In the healthcare setting, the N95 respirator is the most common respirator,^[^ ^20^ ^]^ but despite the significantly greater protection afforded to the user, surgical masks are more frequently used in clinical settings. Surgical masks only offer protection from large particle droplets and fail to shield the user from inhaled small particle droplets, fumes, or vapors^[^ ^29^ ^]^ and do not receive an OSHA APF. Respirators are the only type of PPE that protect against airborne—rather than droplet—hazards; therefore, it is essential that healthcare workers understand the distinctions between surgical masks and respirators and choose the correct form of PPE based on their environmental conditions and assessed risks. One study interviewed 10383 healthcare workers about their use of respirators for various common occupational hazards including: administration of aerosolized drugs, use of chemical sterilants or high‐level disinfectants, treatment of patients with influenza‐like illness, and exposure to surgical smoke. Eighteen percent (18%) of the interviewed healthcare workers reported using a respirator—N95 being the most common at 93% of those reporting respirator use—for one or more of the listed hazards, with administration of aerosolized medications being the most common reason for use of a respirator. The use of surgical masks was significantly more common, with 78% of healthcare workers reporting use of a surgical mask for one or more of the listed hazards. The most frequent reasons for not using respirator protection was that use of respirators was either not part of their institutional protocol or that exposure was expected to be minimal.^[^ ^30^ ^]^ The relative lack of use of respirators for potential chemical hazards represents an opportunity for improvement in the protection against occupational hazards in the healthcare industry.

Although widely utilized in many other industries, reusable EHFRs are used in the healthcare industry significantly less frequently than FFRs.^[^ ^31^ ^]^ In fact, many clinical workers prefer N95 respirators to EHFRs and PAPRs, citing several advantages, including ease of communication and comfort, although users report a greater sense of perceived protection when wearing EHFRs.^[^ ^31^ ^]^ Despite a preference for FFRs in the healthcare community, the increased utilization of EHFRs in the industry may help alleviate shortages of FFRs during strains on the respiratory supply chain.^[^ ^32^ ^]^ That being said, storage and access to these devices continue to be the greatest roadblocks to widespread adoption of EHFRs.^[^ ^33^ ^]^

Recent advancements in elastomeric respirator design have resulted in a class of devices that feature high filtration efficiency, low breathing resistance, and optimal user comfort. The effectiveness of an EHFR is dependent on proper use by the user, and generating a tight seal between the respirator and the wearers face during use, as well as the performance of the filter material.^[^ ^4^ ^]^ As such, annual fit testing and maintenance of worn‐out or broken respirators are essential for ensuring optimal performance. Many EFHRs are designed to form a tight seal around the face and have adjustable straps for achieving optimal fit. Since EHFRs are reusable, they must be cleaned and disinfected in between use. The labor and logistics involved in cleaning these devices at hospitals and other clinical settings may make their use less attractive than FFRs, which do not need to be disinfected as they are disposable. Additionally, the use of EFHRs is more cost‐intensive, as each employee requires their own individual respirator, and the parts and materials involved in manufacturing EFHRs are more costly than FFRs. However, when fitted and maintained correctly, EFHRs can provide greater respiratory protection than FFRs and a greater perceived sense of safety, especially in particularly hazardous environments.

Using various types of respirators, including FFRs, EHFRs, and PAPRs, may be the most effective strategy for respiratory protection in the healthcare industry.^[^ ^34^ ^]^ Surges and strains on the PPE supply chain during emergency situations present challenges in maintaining adequate supply of PPE for clinical staff. Despite their popularity, gradual replacement of single‐use N95s and other FFRs with reusable EHFRs may prove to be a successful solution to PPE shortages even with the added labor required for disinfection. Additionally, the development of a new class of reusable N95 masks adequate for use in clinical settings may provide the optimal balance of user comfort, respiratory protection, and logistical ease for hospitals. These reusable, sterilizable, elastomeric N95 respirators with replaceable filter cartridges were successfully fit tested by 60 healthcare workers across two institutions following an OSHA‐approved testing method. Additionally, users responded favorably when asked about quality of fit, breathability, and ease of replacing the filters; a majority of users also indicated a preference for wearing the reusable respirator over a standard hospital‐issued respirator.^[^ ^26^ ^]^ Furthermore, these respirators showed minimal changes in elasticity following rigorous decontamination in an autoclave, microwave, under UV light, and when exposed to isopropyl alcohol and bleach.^[^ ^27^ ^]^ These reusable N95 respirators eliminate the need for large quantities of disposable N95 respirators and offer simpler disinfection strategies than EHFRs, which may help eliminate the cost, time, and labor associated with current hospital respiratory protection strategies.

## 5 What Needs to Be Filtered?

The shape, size, composition, and concentration of aerosolized hazards vary greatly based on the type of substance and environmental conditions. A careful understanding of these particle characteristics is essential for designing effective respirators and optimally protecting users. In the healthcare setting, the greatest hazard warranting protection is the transmission of aerosolized pathogens generated by the coughing, sneezing, or breathing of infected individuals. These pathogens include bacteria, viruses, and fungi, which vary in their transmissibility, virulence, and viability as airborne particles.^[^ ^35^ ^]^ The diameter of generated particles affects their behavior, aerodynamics, and penetrability. Small aerosols with a diameter of ≤5 µm are often unaffected by the forces of gravity and are therefore able to remain airborne nearly indefinitely unless removed by circulating air.^[^ ^36^ ^]^ As a result, particles of this size are capable of long‐range transmission. Aerosolized pathogens encapsulated within a droplet ≤5 µm in diameter are readily able to penetrate into the lower respiratory tract, yet particles of this size are often exhaled, particularly those <1 µm in diameter.^[^ ^37^ ^]^ Intermediately sized particles between 5–10 µm in diameter are still easily able to travel past the glottis.^[^ ^38^ ^]^ Large aerosols with particle diameters of ≥20 µm are more greatly influenced by the force of gravity and often settle quickly, making long‐range transmission less common. In fact, the average size of settled particles within 2 m of a human sneeze is in the range of 60–100 µm.^[^ ^39^ ^]^ These particles are still able to cause transmission and frequently contain a greater amount of pathogenic material; however, they are not readily able to penetrate into the lower respiratory tract and only reach the upper respiratory tract through short‐range transmission.^[^ ^38^ ^]^ The Infection Diseases Society of America (ISDA) defines particles with diameter ≤10 µm as “respirable,” since they can penetrate into the lower respiratory tract, and particles between 10–100 µm diameter as “inspirable,” since they are confined to the upper respiratory tract.^[^ ^40^ ^]^

Sneezing and coughing result in the generation of plumes of aerosols with a high concentration of particles that gradually travel outward from the source, up to a distance of 7–8 m.^[^ ^41^ ^]^ Although the size of particles in these plumes vary widely,^[^ ^42^ ^]^ a review of several coughing studies of patients with numerous respiratory infections including *Mycobacterium tuberculosis*, *Pseudomonas aeruginosa*, and Influenza A and B consistently found infectious pathogens within small‐sized particles with a diameter <5 µm.^[^ ^43^ ^]^ Exhaled breath studies that included measurements of the above pathogens as well as other mixed viruses similarly identified infectious agents to be within particles with diameters <5 µm.^[^ ^43^ ^]^ Furthermore, an analysis of exhaled particles from influenza‐positive individuals showed that particles ≤5 µm in diameter contained an 8.8‐fold greater viral load than particles >5 µm in diameter.^[^ ^44^ ^]^ Therefore, infected individuals who sneeze or cough are not only able to generate “respirable” particles that can transmit disease across a wide area, but also create particles that can penetrate the lower respiratory tract just through the act of breathing, which can infect others through short‐range transmission.

Since particles with diameters of ≤5 µm have the potential to both travel long distances and penetrate the lower respiratory tract, special attention should be paid to filtering particles of this size. A study that explored the ability of different mask types to filter particles of various sizes found that surgical masks were able to achieve up to a 92% reduction in small‐scale particle emission during breathing, talking, and coughing.^[^ ^45^ ^]^ Although surgical masks may be able to mitigate the majority of aerosolized pathogen transmission, the use of more effective respirators has the potential to further reduce potential exposures, especially in high‐risk individuals such as healthcare workers. In a 2006 performance comparison study, several models of elastomeric N95 respirators, N95 filtering‐face piece respirators, and surgical masks were subjected to six simulated workplace tests by 25 healthcare workers. Based on the exposures experienced by the users, a simulated workplace protection factor (SWPF) was developed to quantify the protection afforded by each device. The results indicated significant differences between the three groups, with elastomeric N95 respirators achieving the greatest median SWPF of 36, followed by N95 filtering‐face piece respirators at 21, and surgical masks at 3, demonstrating that N95 respirators provide significantly enhanced protection relative to surgical masks.^[^ ^46^ ^]^

## 6 Filtration Mechanisms

**Figure 2:**
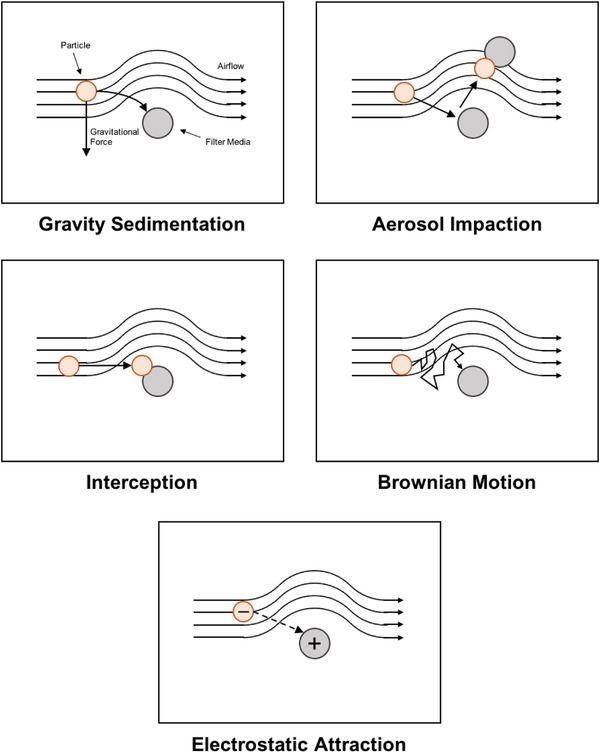
Visualization of several common filtration mechanisms.

Respirator filtration efficiency is highly dependent on the composition, design, and layering of filter media. The wide range of available filter media differentially interacts with particulate matter through various filtration mechanisms. Most filters are designed to strain large particles through small openings between media membranes. However, this method does not contribute to the entire filtration potential of a material, which is highly dependent on particle size, media spacing, and media density. Five additional commonly identified filtration mechanisms include: gravity sedimentation, inertial impaction, interception, diffusion, and electrostatic attraction (**Figure** **2**).^[^ ^47^, ^48^ ^]^ A careful understanding of the various filtration mechanisms employed by filter media is essential for designing an efficient respirator.^[^ ^49^ ^]^

Gravity sedimentation decreases the concentration of particles in the air as particles and aerosols are pulled downward to settle onto the filter.^[^ ^50^ ^]^ Large aerosolized particles (1 to 10 µm) are more greatly affected by gravity sedimentation since the gravitational force on a particle is proportional to its mass.^[^ ^51^ ^]^

Aerosol impaction harnesses the property of inertia to remove particles from the air by forcing aerosols to change direction which causes larger particles to collide with filter media.^[^ ^47^ ^]^ The effectiveness of this filtration mechanism is directly related to the inertia of particles; those with larger diameters, densities, and velocities experience the greatest inertial impaction. Inertial impaction has an effect on particles greater than 1 µm, but does not influence particles with smaller diameters.^[^ ^52^ ^]^

Interception is a mechanism in which low‐inertia particles contact and adhere to filter media, resulting in their removal from the air stream.^[^ ^47^ ^]^ This mechanism differs from inertial impaction in that the particles are captured, rather than deflected, by the filter media. This method is most effective in filtering low‐inertia particles with small diameters, densities, and velocities, as their minimal momentum and inertial forces are unable to overcome the collision with the filter media. Therefore, interception is effective at capturing aerosolized particles with diameters in the range of 100 nm to 1 µm.^[^ ^51^, ^52^ ^]^ The mechanisms of inertial impaction and interception are both highly dependent on the density and composition of the filter media. Media that facilitates a greater number of collisions between particles increases the effectiveness of these mechanisms of filtration.

Brownian motion is the random movement of particles suspended in a liquid or gas as they experience continuous collisions with their surroundings. As these collisions occur, the particles diffuse from areas of higher concentration to areas of lower concentration as described by Fick's law, which says that rate of mass flow per area, or flux, is directly proportional to both the particle concentration gradient and the diffusion coefficient, or diffusivity, of the ambient material. Brownian motion can be leveraged to capture Particles with small diameters that are resistant to gravity sedimentation, inertial impaction, and interception can be captured as a result of collisions secondary to Brownian motion. Filter media with dense matrices and low diffusion coefficients increase the incidence of collisions, preventing the escape of these particles from the media, effectively capturing them as the mass flux through the material is so small.^[^ ^47^ ^]^ This mechanism is most effective at capturing nanoparticles and other particles with diameters less than 0.2 µm,^[^ ^52^ ^]^ but has a significant effect on particles up to 1 µm.^[^ ^51^ ^]^

Electrostatic interactions describe the attractive and repulsive forces between complete or partial ionic species. Harnessing the attractive forces between charged molecules have numerous potential applications from drug delivery^[^ ^53^ ^]^ to respirator design.^[^ ^54^ ^]^ As most particulate matter contains a net charge, the use of electrically charged filter media can effectively filter aerosols through electrostatic deposition, which is the collection of particles on filter media as a result of charge‐based interactions. Electrostatic filter media is manufactured in a way that takes advantage of naturally occurring phenomenon, including induction, the Corona Effect,^[^ ^55^ ^]^ and the triboelectric effect,^[^ ^56^ ^]^ to generate a voltage across synthetic fibers. The effectiveness of electrostatic interactions as a filtration mechanism is dependent on the surface area available for electrostatic deposition: decreasing the diameter of the fibers within the filter media increases the number of fibers that can be used. Electrostatically charged filter media can redirect the path of traveling particles off their streamline onto the fibers, effectively removing them from the airstream.

The numerous filtration mechanisms outlined in this section—gravity sedimentation, inertial impaction, interception, diffusion, and electrostatic attraction—provide a diverse set of potential strategies for aerosol and particle filtration. Although discussed as discrete mechanisms, most filter media employ several of these mechanisms simultaneously to more effectively filter a wide range of particles of various shapes and sizes. In order to employ the use of all of these mechanisms, careful selection and layering of filter media that utilize specific strategies is essential for designing a respirator that is effective in a particular environment.

## 7 Measuring Respirator Performance

**Figure 3:**
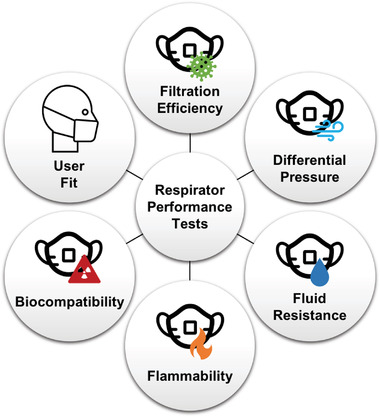
Tests performed to evaluate respirator performance.

Respirators are evaluated through a number of diagnostic assays to determine performance and integrity (**Figure** **3**). Regulatory agencies like NIOSH and the FDA perform standardized tests as part of the certification and approval process (**Table** **1**). These testing protocols evaluate several respirator properties including filtration efficiency, differential pressure drop, fluid resistance, flammability, material biocompatibility, and fit. NIOSH approval requires the evaluation of respirator filtration efficiency and airflow resistance.^[^ ^19^ ^]^ FDA guidance documents^[^ ^57^, ^58^ ^]^ state that in order to achieve FDA approval, PPE must be evaluated for fluid resistance, filtration efficiency, air exchange (or differential pressure between the user and the environment), flammability, and biocompatibility. A careful understanding of these protocols is essential to develop respirators with the desired performance characteristics.

**Table 1:** Standards for the evaluation of respirator performance characteristics

Respirator performance characteristic	NIOSH standard	FDA standard
Overall respirator approval	42 CFR Part 84^[^ ^19^ ^]^	Guidance for Industry and FDA Staff Surgical Masks – Premarket Notification [510(k)] Submissions^[^ ^57^ ^]^ 21 CFR 878.4040(b) class II^[^ ^58^ ^]^
Filtration efficiency	42 CFR Part 84^[^ ^19^ ^]^	PFE: ASTM F2299^[^ ^60^ ^]^ BFE: ASTM F2101^[^ ^61^ ^]^
Differential pressure drop	42 CFR Part 84^[^ ^19^ ^]^	MIL‐M‐36945C 4.4.1.1.1^[^ ^66^ ^]^
Fluid resistance	42 CFR Part 84^[^ ^19^ ^]^	ASTM F1862^[^ ^68^ ^]^
Flammability	N/A	16 CFR Part 1610^[^ ^70^ ^]^
Biocompatibility	N/A	ISO‐10993^[^ ^71^ ^]^
User fit	RCT‐APR‐STP‐0067^[^ ^73^ ^]^	N/A

Filtration efficiency is a measure of the degree to which a filter is able to prevent the passage of aerosolized particulates from the ambient environment to the air delivered to the user. This respirator characteristic is dependent on the material, shape, and layering of filter media, as well as environmental conditions. Typically, high surface area filters with many layers of high‐density material result in the highest filtration efficiency. However, environmental conditions such as temperature and humidity can impair filter function. Furthermore, the particle sizes and composition of aerosols vary widely across settings; as such, respirator filtration efficiency can fluctuate according to the type of aerosol being filtered. In order to account for the diversity of aerosols and environmental conditions a respirator may encounter, regulatory agencies have developed a number of different testing protocols to determine filtration efficiency across conditions.

Aerosol type is an important factor that affects respirator efficiency, since aerosolized particles, bacteria, and viruses each differing significantly in size and composition. As such, different testing protocols exist to measure respirator PFE, bacterial filtration efficiency (BFE), and viral filtration efficiency.^[^ ^59^ ^]^

NIOSH evaluates PFE for N class respirators by measuring the penetration of minute, charge neutralized, polydisperse sodium chloride (NaCl) aerosols and evaluates R and P class respirators using dioctyl phthalate (DOP), a viscous lipid‐soluble liquid.^[^ ^19^ ^]^ Prior to testing, twenty filters are pretreated at 85% relative humidity (RH) and a temperature of 38 °C for 25 h, then sealed in a gas‐tight chamber for an additional 10 h. Then, these experimental filters are exposed to a flow rate of 85 L min^−1^ of the respective aerosolized compound, in 25 °C and 30% RH conditions, with a maximum aerosol concentration of 200 mg m^−3^. This procedure continues until the minimum filtration efficiency has been achieved or 200 mg of the aerosolized material has made contact with the filter. The filtration efficiency is determined by comparing the mass of particulate collected by the filter to the mass of particulate that passed through the filter. The small NaCl (average of 0.075 ± 0.02 µm) and DOP (average of 0.185 ± 0.02 µm) particle diameter, high face velocity, and extreme environmental conditions of this procedure represent maximum penetration and a “worst‐case” scenario; therefore, ensuring effective respirator filtration under less harsh and more realistic conditions. The NIOSH certifications of 95, 99, and 100 correspond to filtration efficiencies of at least 95%, 99%, and 99.97% respectively from this assay.

The FDA specifies a different method of measuring PFE for those devices not receiving NIOSH certification, using 0.1 µm un‐neutralized, polystyrene latex particles at a face velocity of 0.5 to 25 cm sec^−1^.^[^ ^60^ ^]^ In the approval of surgical N95 respirators, the FDA accepts NIOSH PFE testing. Additionally, data for BFE is frequently included in FDA premarket authorizations for surgical N95 respirators.^[^ ^57^ ^]^ The FDA does not perform any testing directly but sets standards for respirator developers, manufacturers, and third‐party evaluators to follow. The FDA BFE test recommends the use of un‐neutralized, *Staphylococcus aureus* aerosol droplets with a mean diameter of 3 µm and a face velocity of 28.3 L min^−1^.^[^ ^61^ ^]^ Determining PFE and BFE enables the evaluation of respirators under a range of potential environmental conditions and aids in the modification of these devices for optimal performance.

Differential pressure drop is a measure of the resistance that airflow experiences while passing through filter media. This important respirator characteristic corresponds to the breathability of the filter, with higher pressure differences making breathing more difficult for the user.^[^ ^62^ ^]^ Therefore, lower pressure drops are desirable to increase breathability and user comfort. Breathing involves both inspiratory and expiratory components and the pressure drop associated with each action must be considered. A larger filter surface area contributes to a decrease in pressure drop and a decrease in face velocity, which improves breathability as the airflow experiences decreased resistance and lower turbulence. Therefore, respirator designs that minimize pressure drop and face velocity should be prioritized.

Differential pressure drop and respirator breathability are highly dependent on environmental conditions. Increases in RH and decreases in external temperature have been shown to affect respiratory breathability, with no effect on particle penetration or most penetrating particle size.^[^ ^63^ ^]^ One study concluded that an increase in RH from 50% to 95% significantly increased pressure drop in a P100 respirator, with no effect on the pressure drop of an N95 respirator examined under the same conditions.^[^ ^64^ ^]^ It is hypothesized that particulate matter experiences greater adhesive forces at a higher RH, causing the formation of chain‐like structures.^[^ ^65^ ^]^ These aerosol chains block the flow of air through the respirator, increasing pressure drop while decreasing breathability and user comfort. It is possible that the differing filter media of the P100 respirator, with a greater filtration efficiency and oil resistance relative to the N95 respirator, exacerbates the adhesive forces of inspired particles resulting in a greater pressure drop and reduced breathability. Although the effects of RH on pressure drop and breathability vary based on respirator type, working in high humidity environments should be avoided when the use of respirators is required to maintain safety and user comfort.

NIOSH evaluates airflow resistance as part of their respirator approval and certification process.^[^ ^19^ ^]^ These guidelines establish maximum pressures for both inhalation and exhalation. To evaluate airflow resistance, the intact experimental respirator is attached to a tight‐fitting testing device and subject to continuous airflow at 85 L min^−1^. NIOSH specifies that the maximum resistance for air‐purifying particulate respirators during inhalation be no greater than 35 mm H_2_O, no greater than 25 mm H_2_O during exhalation, and experience a maximum leakage of no more than 30 mL per minute. These parameters must be achieved by an experimental respirator in order to receive a NIOSH certification and classification. The FDA does not require a specific procedure for the evaluation of pressure drop but suggests certain standards be followed.^[^ ^66^ ^]^ However, the FDA does specify that when reporting the airflow resistance of a device the face velocity, sample size, and flow rates of the testing conditions be described.

Fluid resistance refers to a respirator's ability to resist changes in filtration efficiency when exposed to fluids. NIOSH evaluates the effect of oil contamination on filter efficiency degradation to differentiate between N, R, and P series respirators. N series respirators are not resistant to oil and cannot be used when oil hazards are present in the environment. To classify respirators as R, resistant to oil, or P, oil proof, NIOSH evaluates the filtration efficiency of these devices using DOP, a chemical that significantly affects filtration efficiency, as previously described. R series respirators are classified based on their minimum filtration efficiency after exposure to 200 mg of DOP whereas P series respirators are exposed to continuous DOP until filtration efficiency is stabilized. As P series respirators are able to protect against a greater loading capacity of oil‐based compounds, these respirators can be used for extended periods of time in environments with aerosolized oils limited by considerations of hygiene, damage, and breathing resistance. However, R series respirators may only be used for one work shift, or a maximum of 8 h, in aerosolized oil environments prior to replacement.^[^ ^67^ ^]^ The FDA defines fluid resistance as the ability to resist the penetration of blood and bodily fluids. The recommended procedure evaluates the ability of devices to resist the penetration of a red‐dyed synthetic blood compound across the range of human blood pressures, 80, 120, and 160 mmHg.^[^ ^68^ ^]^ Devices that can resist penetration at greater pressures are more fluid resistant.

Characterization of material flammability and biocompatibility are specific to FDA evaluation. As there are many potential sources of ignition in the clinical setting, the FDA requires the use of NFPA Class 1 and 2^[^ ^69^ ^]^ flammability materials only, which experience only low or intermediate flammability.^[^ ^70^ ^]^ Additionally, because respirators and masks experience prolonged contact with skin, the FDA recommends the evaluation of material biocompatibility.^[^ ^71^ ^]^

The effectiveness of any respirator is dependent on a proper fit, which ensures that all air is forced through the filtering material, rather than being able to leak in from the sides which can result in user exposure to environmental hazards. OHSA guidelines stipulate that in any workplace where respirators are necessary to protect the health of the employees, known as a “hazardous environment”, a written workplace respiratory protection program must be developed with location‐specific procedures.^[^ ^72^ ^]^ This respiratory protection program must include a description of identified hazards and selection of the appropriate respirator for the environment, a framework to ensure the necessary supply of respiratory protection, and procedures to clean, store, and maintain respirators so that they do not present a hazard to the user. Employees are required to comply with the respiratory protection plan, which includes required fit tests annually or whenever a new designation of respiratory is used.^[^ ^16^ ^]^ OSHA and NIOSH outline qualitative fit test protocols using user‐perceived odors and tastes to determine the effectiveness of respirator fit.^[^ ^16^, ^71^ ^]^ Additional methods for assessing proper fit using sensors incorporated into the respirator are under development.^[^ ^27^ ^]^

Isoamyl acetate (IAA) is used as an odor test for non‐particulate respirators. In this protocol, the user is exposed to the banana‐like odor of IAA without a respirator. Then, the user dons an appropriate respirator and enters the testing room, where a paper towel containing IAA is placed above the user. If at any point during a 2‐min time period the user detects the odor of IAA, the test is failed. The user may redon a different respirator and retry after 5 min.

For particulate respirators, a saccharin or Bitrex (denatonium benzoate) solution aerosol taste test is performed. Saccharin is a sweet‐tasting synthetic compound, while Bitrex is a taste aversion agent commonly added to hazardous household liquids. Each taste‐based fit test follows the same procedure. Without a respirator, the user is exposed to the selected aerosolized substance via a nebulizer administered by the testing personnel. The user receives 10 sprays, and if a taste is detected, the taste threshold is set at 10 sprays. If the taste is not detected, 10 more sprays are administered, up to 30 total sprays, with the taste threshold set at the number of sprays required for the user to detect the taste. If after 30 sprays the user cannot detect the taste of the compound, an alternative agent must be used. Then, after donning an appropriate respirator, the user is exposed to the same number of sprays as required to garner a response during the testing phase. Every 30 seconds, the aerosol concentration is replenished using one half the original number of sprays used initially. If at any time the user detects the experimental compound, the test is failed, and the user may retry using a different respirator.^[^ ^73^ ^]^

In addition to selecting the appropriate respirator for the environmental hazards, a user is exposed to, ensuring correct fit is the most essential component of respirator effectiveness and protection. Improper fit or failed fit tests can decrease the protective ability of respirators and place users at greater risk of exposure to hazards. One study of four different N95 respirators examined the APF for users who passed and failed fit tests. Overall, the APF was 1.4 times higher for users who passed a fit test compared to those that did not.^[^ ^74^ ^]^ Similarly, an evaluation of 21 different N95 respirators by NIOSH found that wearing a respirator without fit testing reduced exposures to an average of 33% of the ambient air level, while wearing a respirator after fit testing reduced this exposure to only 4%.^[^ ^75^ ^]^ Therefore, it is evident that a proper respirator fit is essential for ensuring worker protection.

## 8 Material Processing and Relative Performance

Due to the multitude of characteristics needed to satisfy performance and economic demands of respirator filter media, optimized multi‐layer structures consisting of 2D randomly oriented fibers are commonly found. This random orientation is referred to as a nonwoven and may be produced with a number of manufacturing methods which result in a variety of fiber diameters and pore sizes.^[^ ^76^ ^]^

**Figure 4:**
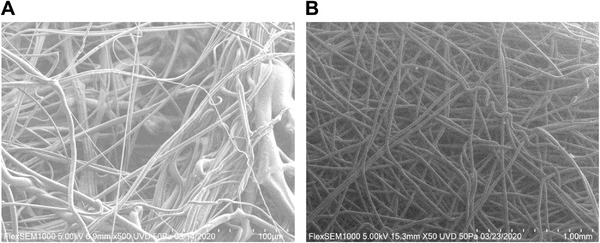
A. Melt‐blown filter media at 500× magnification on scanning electron microscope (SEM). B) Spun‐bond filter media at 50× magnification on SEM.

The nonwoven fabrics are often defined by their manufacturing process, material, basis weight (density in grams per square meter (gsm)), and thickness. Spunbond and melt‐blown are the most commonly used processes produced by different speeds and movement of the fibers during hot‐melt extrusion.^[^ ^77^ ^]^ Spunbond results in larger fibers that filter larger particles, are easy to handle with some durability, and also resist fluid penetration. Melt‐blown fibers are one order of magnitude smaller in diameter, resulting in higher filtration efficiency but must be handled delicately, which is why they are frequently formed on top of spunbond forming the basis of layered spun‐melt‐spun (SMS) fabrics (**Figure** **4**).

Electrostatic filtration offers the best combination of filtration efficiency, breathability, and loading capacity due to the ability to capture smaller particles with larger pores than mechanical filters. The melt‐blown filter media is negatively charged in the manufacturing process via the Corona Effect,^[^ ^55^ ^]^ which imparts a high voltage charge onto the synthetic fibers, providing effective electrostatic particle filtration in the size range of 0.05–5 µm. However, this electrostatic filtration is negatively affected by moisture and environmental relative humidity, giving another reason to encapsulate the delicate material between hydrophobic layers of spunbond. Polypropylene is the material of choice due to its hydrophobicity, low melting temperature, and faster processing speeds leading to better economic value for disposable filters used in healthcare. Novel materials incorporating nanofibers, silver and copper nanoparticles, as well as other ultrafiltration mechanisms are currently under investigation.^[^ ^78^ ^]^

## 9 Clinical Translation and Efficacy

The CDC, OSHA, and IDSA have established guidelines for N95 respirator use in hospitals to reduce infection and transmission of highly pathogenic organisms like *Mycobacterium tuberculosis*.^[^ ^79^ ^]^ N95 respirators have been indicated for use in high to very high‐risk procedures and patient interactions, including patient encounters that have or are suspected of pathogenic viral infections or aerosol‐generating procedures.^[^ ^80^ ^]^ In fact, OSHA requires clinical settings that put healthcare workers at risk for exposure to airborne hazards to institute a respiratory protection program. A comprehensive respiratory protection program must include a procedure for selecting adequate respirators for the workplace, evaluation of respirator fit, procedures for use of respirators in routine and emergency situations, guidelines for disinfection and storage of adequate respirator supplies, and mitigation of airborne hazards through engineering and administrative controls, when available.^[^ ^16^ ^]^

Despite the aforementioned benefits of N95 respirators in filtration and protection, there are conflicting clinical reports on protection against certain viral pathogens compared to standard face masks.^[^ ^81^, ^82^, ^83^ ^]^ One recent prospective cluster‐randomized controlled trial of N95 respirators versus surgical masks in the outpatient setting failed to show a difference in the incidence of influenza. The study enrolled 2862 providers among 360 different cluster settings and evaluated laboratory‐proven influenza through four consecutive influenza seasons. The clusters included diverse outpatient settings of various adult and pediatric medicine clinics and dental clinics that were considered high risk for respiratory illnesses. The study identified 207 laboratory‐positive influenza cases in the N95 FFR group and 193 laboratory‐positive influenza cases in the surgical mask group (95% CI, −0.5% to 2.5%]; *P*  =  0.18).^[^ ^84^ ^]^ Criticisms of the study involved the exposure assessment, including the disease burden of the patients encountered, exposure intensity, or viral infectivity.^[^ ^85^ ^]^ Subsequent meta‐analysis of six clinical trials comparing the effectiveness of N95 respirators and surgical masks found that there was insufficient data to conclude superiority or lack thereof.^[^ ^86^ ^]^

Another important aspect of the N95 respirators is the duration of use. In most clinical situations, healthcare workers will don and doff the N95 respirator as needed for a patient encounter. However, for the greatest degree of protection from viral respiratory illnesses, it was found that continuous use was necessary.^[^ ^87^ ^]^ This is particularly important during respiratory viral seasons and viral pandemics.

Healthcare worker attitudes towards N95 respirators are usually favorable. Most healthcare workers felt more protected using an N95 respirator compared to surgical masks.^[^ ^3^ ^]^ Moreover, two‐thirds of physicians from the IDSA's Emerging Infectious Network preferred N95 respirators over surgical masks during an influenza pandemic despite guidance for use of surgical masks for patients suspected of influenza.^[^ ^88^ ^]^ In a survey performed at a US‐based tertiary care hospital, healthcare workers were asked to assess comfort for N95 respirators at their hospital (3M 1860 model, Kimberly Clark PFR95, and 3M 1870 models) compared to surgical masks using a Likert scale (0–20), and the mean reported comfort score was 13.6 ± 4.9, favoring the N95 respirators.^[^ ^3^ ^]^ During critical shortages of N95 respirators, hospitals have been pushed to reuse their existing N95 respirator supplies. Approximately 53% of users at another US‐based hospital were comfortable with reusing disposable N95 respirators. However, support for reuse strategies was below 40% in general.^[^ ^89^ ^]^

There are major barriers in communication and emotional awareness between patients and clinicians when both parties are wearing N95 respirators and masks. A recent pilot cross‐sectional study among patients, healthcare workers, and healthcare workers who are deaf or hard of hearing evaluated the ability to communicate and perceive facial expressions of a study author wearing transparent and standard surgical masks. Over 75% of study participants were able to identify the emotions expressed when the study author wore transparent masks, whereas less than 25% were able to identify the emotion when the study author wore a standard opaque N95 mask. The majority of respondents felt that communication was improved through the use of a transparent mask.^[^ ^90^ ^]^

## 10 Environmental and Economic Concerns

Respirators are an essential form of PPE for healthcare workers, especially as protection against respiratory illnesses and respiratory transmitted diseases. A Canadian study done by Fraser Health Authority estimated that roughly 5760 disposable respirators (FFRs) are used per month by the healthcare industry.^[^ ^91^ ^]^ Of the respirators that are produced by 3M, the top producer of respirators in the United States, 5 million respirators are dedicated to the healthcare industry per month, approximately 14% of all those produced.^[^ ^92^, ^93^ ^]^ Modeling done by the World Health Organization (WHO) estimates global demand respirators (for countries receiving PPE from WHO) at 35.6 million per month.^[^ ^94^ ^]^

In the context of a global pandemic such as COVID‐19, these estimates increase drastically. In Canada, estimates for respirator demand for healthcare workers during a pandemic increase to approximately 76 000 per month.^[^ ^91^ ^]^ The production of respirators for healthcare workers required by 3M in the United States increases to roughly 13.3 million respirators per month.^[^ ^92^ ^]^ The estimates modeled by WHO show that global demand for respirators increases by 40%, requiring roughly 89 million respirators per month.^[^ ^94^ ^]^ Furthermore, production of disposable face masks is expected to continue increasing by 20% annually between 2020 and 2025.^[^ ^95^ ^]^ Therefore, it is important to consider the environmental and economic impact of respirators.

The design and disposable nature of many medical masks and respirators generate a great amount of environmental waste.^[^ ^96^ ^]^ Rates of overall medical waste generation in Northern Jordan and Wuhan, China are estimated at 0.41 and 0.6 kg per patient per day.^[^ ^97^, ^98^ ^]^ These estimates increased to 3.95 and 2.5 kg per day, respectively, during the COVID‐19 pandemic. Estimates for medical waste produced by China increased by sixfold, from 40 tons per day during routine use prior to the COVID‐19 pandemic to 240 tons per day during the height of the pandemic,^[^ ^99^ ^]^ a large portion which can be attributed to respirators.^[^ ^97^ ^]^

A study done by the UCL Plastic Waste Innovation Hub estimates that roughly 66 000 tons of unrecyclable plastic would be generated per year if every person in the United Kingdom^[^ ^96^ ^]^ wore one disposable surgical mask per day if a nationwide universal mask mandate were employed during the COVID‐19 pandemic.^[^ ^100^ ^]^ There are approximately 18 million healthcare workers in the United States, roughly 25% of the United Kingdom's population.^[^ ^101^, ^102^ ^]^ If each healthcare worker were to wear one mask per day, this would generate roughly 16 500 tons of unrecyclable plastic per year. Considering that the plastic materials required for disposable respirators are greater than that of a surgical mask, it is reasonable to assume that this estimate would be much higher for disposable respirators.^[^ ^96^ ^]^ Estimates from a hypothetical influenza pandemic show that the healthcare worker demand for respirators would be at least four times higher than this, further increasing this estimate.^[^ ^103^ ^]^

**Figure 5:**
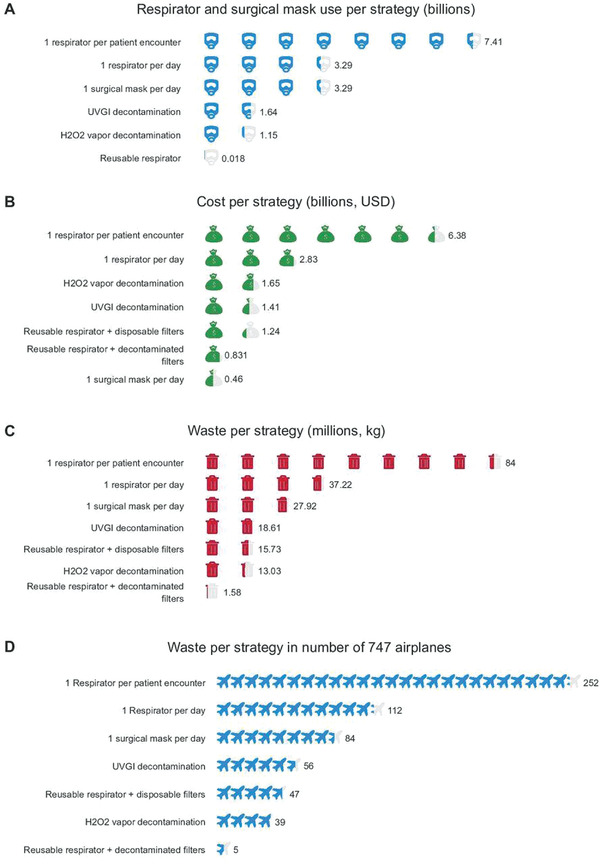
Economic and environmental impacts of respirator use and reuse strategies. Reproduced with permission.^[^ ^104^ ^]^ Copyright 2021, BMJ.

Recent modeling by Chu et al. compared the effect of different respirator extended use and reuse strategies on overall respirator use, cost, and waste generated by the US healthcare industry during the first six months of the COVID‐19 pandemic (**Figure** **5**). This study explored the economic and environmental impact of several respirator use scenarios: 1 single‐use respirator per patient encounter, 1 single‐use respirator per day, 1 single‐use respirator per day following ultraviolet germicidal irradiation or hydrogen peroxide (H_2_O_2_) decamination, 1 reusable respirator per day with disposable or decontaminated filters, and 1 surgical mask per day. Unsurprisingly, the use of 1 respirator per patient encounter was the most cost‐intensive and environmentally hazardous, costing $6.38 billion across six months, while generating 84 million kilograms of waste. The decontamination of single‐use respirators reduced costs and waste generation by 74% and 78% respectively, while the use of a reusable respirator with decontaminated filters decreased costs by 87% and waste generation by 98%. The use of 1 surgical mask per day was the least cost‐intensive strategy, at just $0.46 billion across six months, but generated a significant amount of waste, 27.92 kg.^[^ ^104^ ^]^

The economic impact of respirators in terms of production cost as well as costs to mitigate the negative environmental impact should not be ignored. The global market for PPE increased from approximately $40 billion to $58 billion between 2016 and 2020, roughly a 6.5% compound annual increase.^[^ ^99^ ^]^ Considering the WHO global model which requires 35.6 and 89 million (pre‐COVID setting versus COVID setting) respirators per month for countries worldwide receiving PPE from WHO, monthly costs generated from respirators alone would be upwards of $32 and $81 million, respectively.^[^ ^94^, ^105^ ^]^ Furthermore, inappropriate incineration or disposal of hazardous medical waste in landfills contributes to pollution and waste water run‐off, which is harmful to ecosystems and the environment, contributing to climate change.^[^ ^106^ ^]^ These environmental damages have an additional cost, with an average annual cost due to environmental damages of $0.6 trillion for every year of inaction or delayed mitigation in response to climate change.^[^ ^107^ ^]^

## 11 Conclusion

Respirators provide a high degree of protection from aerosolized agents and are widely used in the healthcare setting. Material selection and respirator design are critical aspects of their performance and use. Respirators are qualified by standards created by NIOSH to provide high levels of protection in clinics and hospitals against the most virulent pathogens. Given their frequency of use, there are significant environmental and economic consequences, especially during times of pandemics.

## Conflict of Interest

J.B., A.J.W., and G.T. are co‐inventors on provisional patent applications of reusable respirators and hold a financial interest in Teal Bio, a biotechnology company focusing on the development of reusable PPE including respirators. Complete details of all relationships for profit and not for profit for G.T. can be found at the following link: https://www.dropbox.com/sh/szi7vnr4a2ajb56/AABs5N5i0q9AfT1IqIJAE-T5a?dl=0.
